# On the Origin and Properties of Liquid Styrax; Extracted from a Memoir Read to the Medical Society of Paris
*"The genuine liquid Styrax is, even at Moco, a very rare commodity, and sold at a very high price, and it has seldom entered the shops of our apothecaries. A resinous juice, possessing somewhat of the same sensible qualities, brought from the Spanish provinces of South America, and perhaps the product of the same tree, is sometimes sold in place of it. But much more frequently what we meet with under this name, is an artificial compound of solid storax, common resin, wine, and oil, beat up together, to a proper consistence. Concerning the real virtues of liquid styrax, observations are altogether wanting, &c."New Edinburgh Dispensatory.


**Published:** 1799-04

**Authors:** Bouillon La-Grange


### *On the Origin and Properties of Liquid Styrax*; extracted from a Memoir read to the Medical Society of Paris; by Citizen* BOUILLON LA-GRANGE.

THE preparation of a medicine can never be made with fuccefs, unlefs the nature of the fubftance of which the mixture is compofed be well underftood. In pharmacy we find a number of chafms of this kind; liquid ftyrax is one of thofe fubjects, on which different conjectures have been formed, and the nature of which has not hitherto been completely developed.

It is well-known, that liquid ftyrax has been employed for ages, in a medicament to which it formerly gave its name, but which now is almoft entirely abandoned, the *unguentum e ftyrace*. It is alfo well known, that the preparation of this ointment varies according to different authors, and that all of them, for want of having a correct knowledge not only of ftyrax, but of the manner in which caloric and oils act on refinous fubftances, have fallen into errors which muft, in general, be attended with an imperfect method of preparing certain plaifters and ointments.

Liquid ftyrax is a refinous juice, called by the Arabians, *Mitia;* by the Turks, *Cotter-miza;* by the Chinefe, *Roca malha;* and by the Europeans, falfe aromatic Storax. This refin is liquid, glutinous, little, or fcarcely at all pellucid, of a grey-brown colour, with the ftrong, but difagreeable fmell of folid ftorax, and of a fomewhat pungent aromatic tafte; it very feldom, if ever, comes to us in a pure unadulterated ftate.

A great diverfity of opinions prevails relative to the origin of this fpecies of refinous balm; fome will have it to be a turpentine, compounded or boiled up with oil, wine, &c.; others maintain, that it is an extract obtained by the decoction of feveral parts of the amber-tree. It fhould be obferved, however, that JAMES PETTIVER, an apothecary of London, and a fkilful naturalift, does not hefitate, in the "*Philofophical Tranfactions*," No. 313, to declare, that it is the juice of a certain tree, called *Rofa malos, (Liquid-ambra*

* "The genuine liquid Styrax is, even at Moco, a very rare commodity, and sold at a very high price, and it has seldom entered the shops of our apothecaries. A resinous juice, possessing somewhat of the same sensible qualities, brought from the Spanish provinces of South America, and perhaps the product of the same tree, is sometimes sold in place of it. But much more frequently what we meet with under this name, is an artificial compound of solid storax, common resin, wine, and oil, beat up together, to a proper consistence. Concerning the real virtues of liquid styrax, observations are altogether wanting, &c."

*New Edinburgh Dispensatory.*

*ambra ſtyraciflua. Lin.*) which grows in the iſle of Cobras, in the Red Sea, about three days journey diſtant from the city of Suez. According to this author, the bark of the tree is peeled off every year; it is afterwards bruiſed or pounded, and boiled in ſea-water to the conſiſtence of glue; then the reſinous ſubſtance which ſwims at the top is collected; it is further purified and refined by diſſolving it again in ſea-water, and ſtraining the ſolution; thus prepared, it is incloſed in ſeparate caſks, together with the reſiduum of the proceſs now deſcribed. Theſe two ſorts of ſtyrax, are then tranſported to Moco and expoſed to ſale at the celebrated Arabian fair.

This perfume, ſays *Valmont Bonare*, is in great eſteem among the Orientals. A caſk containing 420lb. ſells at from 180 to 360 pounds of ſilver, (*livres d'argent*) according to the purity of the ſtyrax. This naturaliſt mentions his having ſeen, in a Turkiſh veſſel, a barrel made of ſtyrax wood, and which, as he was aſſured, had been formed of the trunk of a tree producing the liquid ſtyrax itſelf; the barrel was two feet in diameter; the trunk had been hollowed longitudinally, to the thickneſs or depth of the lower end, the upper end being made of pieces put together: the wood was rather hard, odoriferous, and of a yellowiſh colour. He was further informed, that the ſarcophagi, or mauſolea of great men, were generally made of this ſpecies of wood.

There is, beſides this, another ſubſtance to which the name of ſtyrax has been given; it is imported from America, but is in fact the liquid amber.

This is all that relates to the hiſtory of ſtyrax, among which the account given by Pettiver, appears to be the moſt conſiſtent with the chemical analyſis of that ſubſtance.—As to that which is ſuppoſed to be artificially compounded, it is only the product of fraud or avarice; but it is eaſy to diſtinguiſh the true ſtyrax from that which is manufactured.—The following are the phyſical or ſenſible properties of the liquid ſtyrax uſed as an article of commerce: it is of a greeniſh-grey, the taſte rather aromatic, leaving a ſlight acidity on the tongue. That which exudes from the tree, not having undergone the boiling proceſs we have before deſcribed, and which is called the *true ſtyrax*, is of a deep red colour, of an agreeable ſmell, approaching to that of the balſam of Peru, the taſte pungent, leaving on the tongue a ſmart impreſſion of acidity; this ſpecies is very rare.

The chemical properties of the ſtyrax, which is an object of trade, are too numerous to be inſerted here. From the reſult, however, of its chemical analyſis, the following concluſions may be deduced: 1. That ſtyrax is a reſinous balm, analogous to ſtyrax, or benzoine; 2. That this ſubſtance is compoſed composed of benzoic acid and of a resin, and that the other matters found in it, are not naturally a part of its composition; 3. That the most simple and advantageous method of purifying styrax is, to make a tincture of it in highly-rectified spirit of wine, as it may thus be obtained free from all heterogeneous ingredients; 4. That, in order to prepare the ointment of styrax, and in general, all unguents composed in part of resinous substances, they should not be made too hot, particularly styrax, which is easily decomposed by caloric; as this agent very readily absorbs the oxygen, while the benzoic acid is volatilized at a low temperature. And lastly, that there is a very remarkable difference between the ointment prepared with the styrax purified by alkohol, and that purified by the action of heat; the former has an agreeable flavour of storax, and produces no feculent impurities by its union with other substances; while the latter has a strong, rather disagreeable smell, and always leaves behind a considerable sediment.

